# Antiviral activities of *Radix isatidis* polysaccharide against pseudorabies virus in swine testicle cells

**DOI:** 10.1186/s12906-020-2838-4

**Published:** 2020-02-11

**Authors:** Chao Tong, Zewen Chen, Fang Liu, Yanyan Qiao, Tong Chen, Xuebing Wang

**Affiliations:** 1 College of Animal Science and Veterinary Medicine, Henan Agricultural University, Zhengzhou, 450000 China; 2 Wuhu Overseas Student Pioneer Park, Wuhu, 241006 China; 3 Key Laboratory for Animal-Derived Food Safety of Henan province, Zhengzhou, 450000 China

**Keywords:** *Radix isatidis*, Polysaccharide, Pseudorabies virus, Swine testicle cells

## Abstract

**Background:**

*Radix isatidis* has been used in China and other Asian countries for its antiviral and anti-inflammatory effects for thousands of years. However, the antiviral effect of *Radix isatidis* polysaccharide against pseudorabies virus (PRV) is still unknown.

**Methods:**

The polysaccharide were isolated from extract of the roots of *Radix isatidis.* MTT assays were used to determine the preventive effect, inhibitory effect and antiviral effect of *Radix isatidis* polysaccharide on PRV in vitro.

**Results:**

This study found that different concentrations of polysaccharides from this plant can inhibit PRV replication by 14.674–30.840%, prevent infection at rates of 6.668–14.923%, and kill this virus at rates of 32.214–67.422%.

**Conclusion:**

These results broaden the understanding of this traditional Chinese herb and provide a theoretical basis for further research. Moreover, *Radix isatidis* polysaccharide could be used for antiviral therapy.

## Background

Pseudorabies virus (PRV) is a double-stranded DNA virus and the causative agent of Aujeszky’s disease [1], which is a highly infectious neurological and respiratory disease of pigs. It can lead huge economic losses to the global swine industry as PRV epidemics can lead to an average loss of $6/cwt [2, 3]. PRV has a vast host range and most mammals and some birds can be infected by this pathogen [4]. Swine is the natural reservoir and is susceptible to PRV. After young pigs or other susceptible species are infected, PRV is often lethal and can also lead to central nervous system disorders [5]. Accordingly, it can cause huge economic losses to the swine industry in many countries [6]. Despite the fact that humans are not typically susceptible to infection, one case of PRV resulting in infectious endophthalmitis was reported [7]. Since the first antiviral drug was approved in 1963, many others have been produced for clinical use [8]. However, with respect to clinical therapy for humans or animals, various viral strains are resistant to antiviral drugs [9, 10]. To solve this problem, novel antiviral drugs or compounds are urgently needed.

Herbal medicine has been widely used in China for thousands of years [11, 12] and numerous studies have shown the antiviral, antimicrobial, and anti-inflammatory effects of herbal medicine [13]. For example, *Radix isatidis* (Ban-Lan-Gen) is one famous traditional herbal medicine in China. The dry root (*Radix isatidis*) of the plant *Isatis tinctoria L*. or *Isatis indigotica Fort*. has been used for thousands of years to cure headaches, fever, and sore throats and for detoxification in China. Previous research has shown that *Radix isatidis* has many pharmacological activities including anti-microbial, antiviral, anti-inflammatory and anti-cancer effects [14]. Xiao et al. investigated the extract of *Radix isatidis* and found that it has anti-oxidant and anti-inflammatory activities in vitro [15]. Moreover, Kong et al. found that four organic acids (syringic acid, 2-amino-benzoic acid, salicylic acid, benzoic acid) from *Radix isatidis* could inhibit the growth of *E. coli* [16]. Ma et al. showed that the water extract from *Radix isatidis* has stronger antimicrobial activity against *H. pylori* than other plant extracts [17].

In addition, *Radix isatidis* has obvious antiviral effects. Specifically, it can inhibit the replication of human influenza viruses (H1N1 and H3N2), avian influenza viruses (H6N2 and H9N2) [18], respiratory syncytial virus (RSV) [19], and human herpes simplex virus type I (HSV-1) [20]. Polysaccharides comprise one of the main components of *Radix isatidis* and these also have the anti-oxidant, anti-inflammatory, and antiviral activities [21]. However, the antiviral effect of *Radix isatidis* polysaccharide against PRV is still unknown. Few studies have focused on this point and accordingly, in this study, we explored the antiviral effect of *Radix isatidis* polysaccharide using swine testicle cells and investigated its protective effect against PRV infection.

## Methods

### Preparation of *Radix isatidis* polysaccharide

Thanks to the Henan Province Industry-University-Research Cooperation Project for supporting this research. *Radix isatidis* was purchased from Henan Herbal Medicine Center Chain Co., Ltd. (Zhengzhou, China). A voucher specimen (No. 2013-zz-012) was deposited at the herbarium of laboratory of ethnopharmacology at Henan Agricultural University. The crude herbs were authenticated by Prof. HY Zhang, Department of Chinese Herbal Medicine of Henan Agricultural University. The methods to separate *Radix isatidis* polysaccharide from the plant were in accordance with previous work [22]. Briefly, the fresh roots of *Isatis indigotica Fort*. were cut into small pieces. Approximately 100-g roots were soaked in 600 mL water for 2 h and then boiled at 100 °C for 30 min. The solution was filtered and concentrated under reduced pressure at 50 °C. After the concentrated liquid was precipitated with 60% ethanol at 4 °C, the suspension was collected and concentrated under reduced pressure at 50 °C. Total purified polysaccharide were measured by Vitriol-anthracene ketone and glucose without H2O was tested as standard control. The content of total Radix isatidis polysaccharide was 92.76%. Purified polysaccharides were stored at − 20 °C for further study. All experiments were approved by the Chinese Medicine Research Ethics Committee of Henan Agricultural University (Number HENAUCC1035).

### Virus and cells

The PRV MinA strain was maintained at the Animal Food Safety Key Laboratory (Henan Province, China) [23, 24]. Swine testicle cells were kindly gifted by Dr. Zhanyong Wei (Henan Agricultural University, China). The cells were cultured with RPMI 1640 medium with 10% fetal bovine serum (HyClone, UT, USA), 100 U/mL penicillin, and 100 U/mL streptomycin (HyClone, UT, USA) at 37 °C with 5% CO_2_.

### Determination of *Radix isatidis* polysaccharide cytotoxicity

The cytotoxic effect of *Radix isatidis* polysaccharide toward swine testicle cells was evaluated by performing MTT assays (Sigma-Aldrich, MO, USA) as described previously [25]. Swine testicle cells were digested with trypsin and seeded into 96-well cell culture plates at 5 × 10^5^ cells/well. After culture for 36 h, several different concentrations of *Radix isatidis* polysaccharide were added to each well (repeated four times). Then, cells were cultured for 68 h, MTT reagent was added, and the cell culture plate was incubated at 37 °C with 5% CO_2_ for 4 h. The supernatant was discarded and 100 μL of DMSO was added to each well, which was followed by gentle oscillation for 10 min. The OD values were then measured using a Thermo Scientific Multiskan™ FC microplate reader (Thermo, MA, USA). Each MTT experiment was repeated three times with four wells per point for each concentration.

### Preventive effect of *Radix isatidis* polysaccharide on PRV infection

Swine testicle cells were cultured in 96-well plates as described. After culture for 24 h to achieve 80% confluence, the supernatant was removed and various concentrations of *Radix isatidis* polysaccharide were added to each well. The culture medium with *Radix isatidis* polysaccharide was discarded after a 4-h incubation. Cells were then infected with 100 TCID_50_ (50% tissue culture infective dose) of PRV for 1.5 h. The TCID_50_ was calculated by the Reed-Muench method [25]. Next, cells were incubated at 37 °C with 5% CO_2_ for 68 h. MTT assays were then applied as described previously herein.

### Inhibitory effect of *Radix isatidis* polysaccharide on PRV viral replication

Swine testicle cells were cultured in 96-well plates as described. After culture for 24 h to achieve 80% confluence, cells were infected with 100 TCID_50_ of PRV for 1.5 h. Next, the virus fluid was removed, and cells were washed with PBS three times. Several different concentrations of *Radix isatidis* polysaccharide and RPMI 1640 medium were added. Cells were also incubated at 37 °C with 5% CO_2_ for 68 h and MTT assays were then performed as described.

### Extracellular virucidal assay

Several different concentrations of *Radix isatidis* polysaccharide were mixed together with 100 TCID_50_ of PRV and incubated at 37 °C for 2 h. Then, the mixture was added to each well containing swine testicle cells. After a 24-h incubation, the cell were subjected to an MTT assay as described.

### Statistical analysis

All statistical analysis were performed with SPSS 18.0 (IBM, NY, USA) using a one-way ANOVA. The data are presented as the mean ± SD of quadruplicate independent experiments. A value of *p* < 0.05 was chosen as the criterion of statistical significance.

## Results

### Toxicity of *Radix isatidis* polysaccharide toward swine testicle cells

The cytotoxic effect of *Radix isatidis* polysaccharide extracts on porcine testicle cells was determined by the MTT method after a 68-h co-incubation. The concentrations of *Radix isatidis* polysaccharide tested were 5, 2.5, 1.25, 0.625, 0.3125, 0.15625, 0.078125, and 0.0390625 mg/mL. Results revealed no or insignificant cytotoxic effects of *Radix isatidis* polysaccharide extracts from 0.625 to 0.0390625 mg/mL, with only a slight cytotoxic effect (30.390%) at the highest tested concentration of 5 mg/mL (Table 1). Thus, *Radix isatidis* polysaccharide concentrations of 0.625–0.078125 mg/mL were selected for the next experiments.

**Table 1:** Toxicity of *Radix isatidis* polysaccharide on swine testicle cells

Group	Concentration (mg/mL)	Percentage of inhibition
*Radix isatidis* polysaccharide	5	30.390% ± 0.927
	2.500	19.970% ± 0.838
	1.250	16.800% ± 1.002
	0.625	−1.140% ± 0.406
	0.313	−5.530% ± 1.079
	0.156	−12.060% ± 1.054
	0.078	2.150% ± 1.942
	0.039	3.100% ± 5.331
Negative control	0	0

Note:The experiment was repeated three times for each concentration

### Inhibitory effect of *Radix isatidis* polysaccharide on PRV replication

**Fig. 1:**
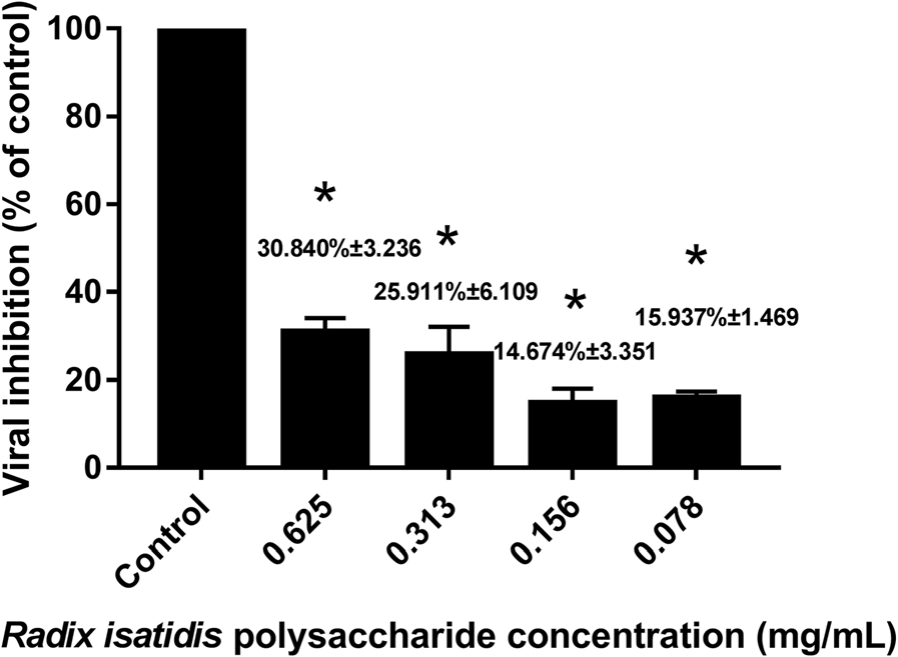
Inhibitory effect of *Radix isatidis* polysaccharide on pseudorabies virus (PRV) replication.* *P* < 0.05 between the control group and the experimental group. Error bars, SD indicate quadruplicate independent experiments. The *Radix isatidis* polysaccharide concentration of the control group was 0 mg/mL in the experiment

To evaluate the effect of *Radix isatidis* polysaccharide on PRV replication, porcine testicle cells were first infected with PRV, which was followed by the addition of various concentrations of *Radix isatidis* polysaccharide. Based on the results, this extract was found to effectively inhibit PRV replication. Figure 1 shows that the rate of inhibition of PRV replication was 14.674–30.840%.

### Preventive effect of *Radix isatidis* polysaccharide on PRV infection

**Fig. 2:**
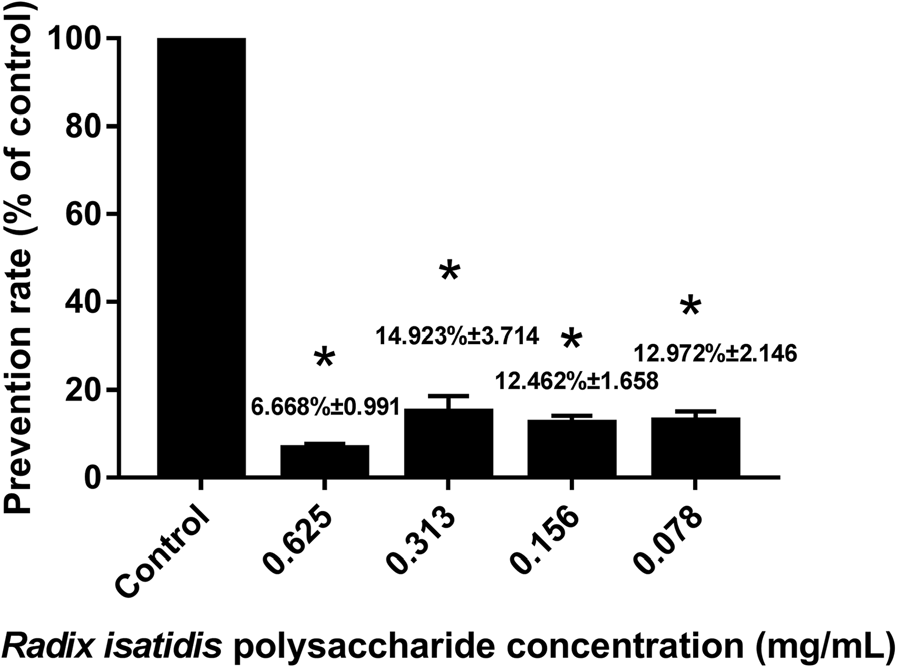
Preventive effect of *Radix isatidis* polysaccharide on pseudorabies virus (PRV). * P < 0.05 between the control group and the experimental group. Error bars, SD indicate quadruplicate independent experiments. The *Radix isatidis* polysaccharide concentration of the control group was 0 mg/mL in the experiment

To evaluate the preventive effect of *Radix isatidis* polysaccharide on PRV infection, porcine testicle cells were first co-cultured with different concentrations of *Radix isatidis* polysaccharide, which was followed by infection with PRV. Results showed that this treatment could prevent PRV infection at rates ranging from 6.668 to 14.923% (Fig. 2).

### Direct effect of *Radix isatidis* polysaccharide on PRV death

**Fig. 3:**
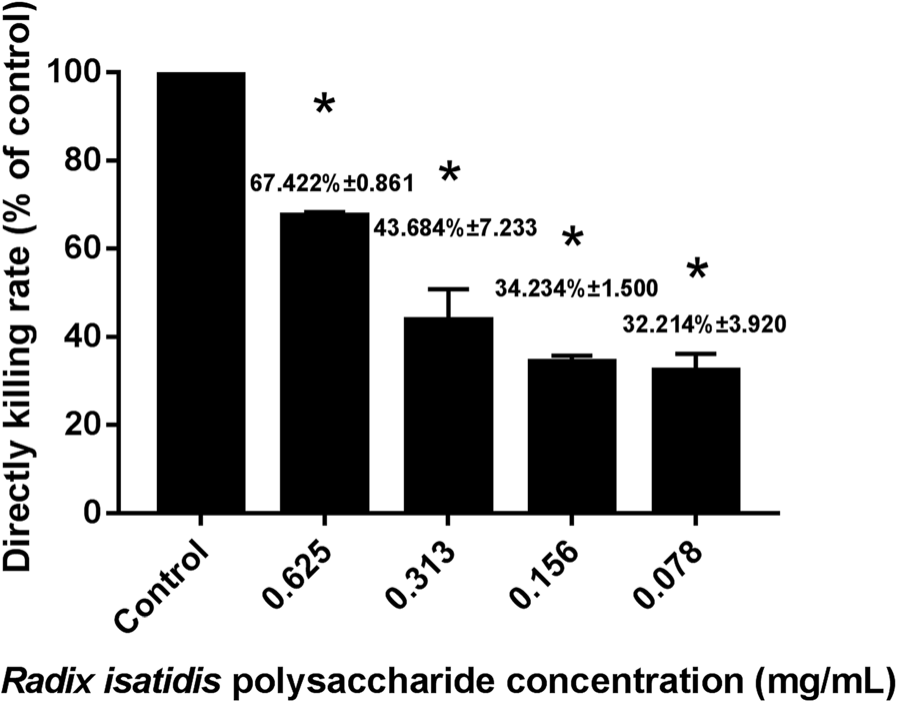
Direct killing effect of *Radix isatidis* polysaccharide on pseudorabies virus (PRV). * P < 0.05 between the control group and the experimental group. Error bars, SD indicate quadruplicate independent experiments. The *Radix isatidis* polysaccharide concentration of the control group was 0 mg/mL in the experiment

The antiviral effect of *Radix isatidis* polysaccharide is of paramount importance for the drug industry. Compared to that in the control group, *Radix isatidis* polysaccharide markedly improved PRV killing. A concentration of 0.625 mg/mL was found to kill 67.422% of PRV, whereas even the lower concentration of 0.078125 mg/mL resulted in 32.214% PRV death (Fig. 3). This indicated that *Radix isatidis* polysaccharide has potential for clinical therapy.

## Discussion

PRV is a neurotropic virus that infects both the peripheral and nervous system. It causes huge economic losses in some domestic and wild animals and is especially devastating for the swine industry [26]. Although PRV has a broad host range, pigs are its natural hosts and serve as a viral reservoir [6]. Humans working on a hog farm can also be infected by PRV [7]. In this study, we reported that *Radix isatidis* polysaccharide can inhibit PRV replication, prevent infection, and kill the virus. Thus, *Radix isatidis* polysaccharide might be adopted for PRV therapy in the future.

Chinese herbal medicines have been reported to inhibit and destroy pathogenic microorganisms. Numerous Chinese herbal medicines have been widely used for the prevention and cure of viral diseases in China and other Asian countries for thousands of years. The newly identified dibenzocyclooctane lignan kadsurindutins A (1) isolated from the stems of *Kadsura induta* was found to exert antiviral effects against Hepatitis B Virus [27]. Moreover, samarangenin B (Sam B), isolated from *Limonium sinense,* has antiviral function and can suppress HSV-1 replication [28]. Moreover, berberine, *Coptidis rhizome*, and Ching-Wei-San have antiviral effects against HSV [29], whereas polyphenolic compounds isolated from *Saxifraga melanocentra* have anti-HCV activity [30]. In total, 11 chemical constituents isolated from *Radix isatidis* have antiviral effects on influenza A1 virus (FM1) and five chemical constituents isolated from *Radix isatidis* have antiviral effects against RSV in vitro [19]. *Radix isatidis* also has antiviral effects on influenza virus, HCMV, HSV-1, and HSV-2 [14]. This study found that the *Radix isatidis* polysaccharide can inhibit PRV infection and kill this virus. These results showed that this extract might contain one or more broad-spectrum antiviral agents. Mechanistically, some of the active chemical constituents or biologically active components of *Radix isatidis* polysaccharide probably enhance protective immunity in response to virus infection. To address this, liquid chromatography is the preferred method for the analysis of compounds from Chinese herbal medicines. The polar and thermally-unstable compounds can be fractionated by capillary electrophoresis [31]. However, the isolation and characterization of highly effective compounds and the accurate determination of the composition of active ingredients from Chinese herbal medicines is still a major problem. After separating these effective components, they can be used not only for PRV, but could also be tested against some human viruses such as human influenza virus or HIV. These effective broad-spectrum antiviral agents might thus provide a new treatment option for human viruses.

## Conclusions

In summary, *Radix isatidis* polysaccharide can inhibit PRV replication, prevent infection, and kill the virus. This study will ultimately broaden the understanding of *Radix isatidis* and provide a theoretical basis for further research. However, more studies are needed to fully explore the mechanism underlying these antiviral effects*.*

## Data Availability

The datasets used and/or analyzed during the current study are available from the corresponding author on reasonable request.

## Abbreviations

FM1: Influenza A1 virus
HSV-1: Human herpes simplex virus type I
PRV: Pseudorabies virus
RSV: Respiratory syncytial virus
TCID_50_: 50% tissue culture infective dose

## References

[1] Mettenleiter TC (2000). Aujeszky's disease (pseudorabies) virus: the virus and molecular pathogenesis--state of the art, June 1999. Vet Res.

[2] Miller GY, Forster DL, Tsai J, Bowman G (1995). Productivity and profitability differences between pseudorabies-infected and pseudorabies-noninfected farrow-to-finish swine herds. J Am Vet Med Assoc.

[3] Miller GY, Tsai JS, Forster DL (1996). Benefit-cost analysis of the national pseudorabies virus eradication program. J Am Vet Med Assoc.

[4] Klupp BG, Hengartner CJ, Mettenleiter TC, Enquist LW (2004). Complete, annotated sequence of the pseudorabies virus genome. J Virol.

[5] Sui X, Yin J, Ren X (2010). Antiviral effect of diammonium glycyrrhizinate and lithium chloride on cell infection by pseudorabies herpesvirus. Antivir Res.

[6] Muller T, Hahn EC, Tottewitz F, Kramer M, Klupp BG, Mettenleiter TC, Freuling C (2011). Pseudorabies virus in wild swine: a global perspective. Arch Virol.

[7] Ai JW, Weng SS, Cheng Q, Cui P, Li YJ, Wu HL, Zhu YM, Xu B, Zhang WH (2018). Human Endophthalmitis caused by Pseudorabies virus infection, China, 2017. Emerg Infect Dis.

[8] De Clercq E, Li G (2016). Approved antiviral drugs over the past 50 years. Clin Microbiol Rev.

[9] Morfin F, Thouvenot D (2003). Herpes simplex virus resistance to antiviral drugs. J Clin Virol.

[10] Zoulim F (2011). Hepatitis B virus resistance to antiviral drugs: where are we going?. Liver Int.

[11] Li T, Peng T (2013). Traditional Chinese herbal medicine as a source of molecules with antiviral activity. Antivir Res.

[12] Pan MH, Chiou YS, Tsai ML, Ho CT (2011). Anti-inflammatory activity of traditional Chinese medicinal herbs. J Tradit Complement Med.

[13] Muluye RA, Bian Y, Alemu PN (2014). Anti-inflammatory and antimicrobial effects of heat-clearing Chinese herbs: a current review. J Tradit Complement Med.

[14] Zhou W, Zhang XY (2013). Research progress of Chinese herbal medicine Radix isatidis (banlangen). Am J Chin Med.

[15] Xiao P, Huang H, Chen J, Li X (2014). In vitro antioxidant and anti-inflammatory activities of Radix Isatidis extract and bioaccessibility of six bioactive compounds after simulated gastro-intestinal digestion. J Ethnopharmacol.

[16] Kong W, Zhao Y, Shan L, Xiao X, Guo W (2008). Thermochemical studies on the quantity-antibacterial effect relationship of four organic acids from Radix Isatidis on Escherichia coli growth. Biol Pharm Bull.

[17] Ma F, Chen Y, Li J, Qing HP, Wang JD, Zhang YL, Long BG, Bai Y (2010). Screening test for anti-helicobacter pylori activity of traditional Chinese herbal medicines. World J Gastroenterol.

[18] Li Zhengtu, Li Li, Zhou Hongxia, Zeng Lijuan, Chen Tingting, Chen Qiaolian, Zhou Beixian, Wang Yutao, Chen Qiaoyan, Hu Ping, Yang Zifeng (2017). Radix isatidis Polysaccharides Inhibit Influenza a Virus and Influenza A Virus-Induced Inflammation via Suppression of Host TLR3 Signaling In Vitro. Molecules.

[19] Ye WY, Li X, Cheng JW (2011). Screening of eleven chemical constituents from Radix Isatidis for antiviral activity. Afr J Pharm Pharmaco.

[20] He LW, Liu HQ, Chen YQ, Yang JY, Wang TL, Li W (2014). Total synthesis and anti-viral activities of an extract of Radix isatidis. Molecules.

[21] Du Z, Liu H, Zhang Z, Li P (2013). Antioxidant and anti-inflammatory activities of Radix Isatidis polysaccharide in murine alveolar macrophages. Int J Biol Macromol.

[22] Wang X, Xue Y, Li Y, Liu F, Yan Y, Zhang H, Jin Q (2018). Effects of Isatis root polysaccharide in mice infected with H3N2 swine influenza virus. Res Vet Sci.

[23] Liu C, Liu Y, Tian Y, Wei X, Zhang Y, Tian F (2018). Genetic characterization and mutation analysis of Qihe547 Aujeszky's disease virus in China. BMC Vet Res.

[24] Wen R, Luo T, Yu K, Huang W (2000). Isolation and identification of two isolates of pseudorabies virus in Guangxi. Guangxi Nongye Shengwu Kexue.

[25] Mosmann T (1983). Rapid colorimetric assay for cellular growth and survival: application to proliferation and cytotoxicity assays. J Immunol Methods.

[26] Enquist Lynn W. (1994). Infection of the mammalian nervous system by pseudorabies virus (PRV). Seminars in Virology.

[27] Ma W, Ma X, Huang H, Zhou P, Chen D (2010). Dibenzocyclooctane Lignans from the stems of Kadsura induta and their antiviral effect on hepatitis B virus. Chem Biodivers.

[28] Kuo YC, Lin LC, Tsai WJ, Chou CJ, Kung SH, Ho YH (2002). Samarangenin B from Limonium sinense suppresses herpes simplex virus type 1 replication in Vero cells by regulation of viral macromolecular synthesis. Antimicrob Agents Ch.

[29] Chin LW, Cheng YW, Lin SS, Lai YY, Lin LY, Chou MY, Chou MC, Yang CC (2010). Anti-herpes simplex virus effects of berberine from Coptidis rhizoma, a major component of a Chinese herbal medicine, Ching-Wei-San. Arch Virol.

[30] Zuo GY, Li ZQ, Chen LR, Xu XJ (2005). In vitro anti-HCV activities of Saxifraga melanocentra and its related polyphenolic compounds. Antivir Chem Chemother.

[31] Ong ES (2004). Extraction methods and chemical standardization of botanicals and herbal preparations. J Chromatogr B Analyt Technol Biomed Life Sci.

